# Clinical research on the efficacy and safety of Bosinji for low back pain with radiculopathy caused by herniated intervertebral disc of the lumbar spine: A protocol for a multicenter, randomized, controlled equivalence trial

**DOI:** 10.1097/MD.0000000000013684

**Published:** 2018-12-14

**Authors:** Bonhyuk Goo, Sung-Jin Kim, Eun-Jung Kim, Dongwoo Nam, Hyun-Jong Lee, Jae-Soo Kim, Yeon-Cheol Park, Yong-Hyeon Baek, Sang-Soo Nam, Byung-Kwan Seo

**Affiliations:** a Department of Acupuncture and Moxibustion, Kyung Hee University Hospital at Gangdong, Seoul; b Department of Acupuncture and Moxibustion Medicine, College of Oriental Medicine, Dongguk University, Gyeongsangbuk-do; c Department of Acupuncture and Moxibustion Medicine, College of Korean Medicine, Kyung Hee University, Seoul; d Department of Acupuncture and Moxibustion medicine, College of Korean medicine, Daegu Haany University, Daegu, South Korea.

**Keywords:** bosinji, herbal medicine, low back pain, lumbar disc herniation, lumbar herniated intervertebral disc, radiculopathy

## Abstract

**Background:**

A lumbar herniated intervertebral disc (LHIVD) is a common problem that usually causes low back pain and radiating pain. The effectiveness of Bosinji, one of the herbal medicines used for low back pain and radiating pain in patient with LHIVD, has been reported in several studies; however, little clinical evidence is available owing to the methodological limitations in previous studies. Hence, the present study aims to establish the clinical evidence regarding the efficacy and safety of Bosinji in improving pain, function, and quality of life in LHIVD patients.

**Method/design:**

This is a multicenter, open-label, randomized, controlled, and equivalence trial with 2 parallel arms. A total of 74 patients who have low back pain and radiating pain due to LHIVD will be recruited and randomly allocated to the experimental group and control group. The patients in the experimental group and control group will take 2.5 g of Bosinji granule (1.523 g of Bosinji extract) or Loxonin tablet (60 mg of loxoprofen) 3 times a day for 6 weeks. Additionally, both groups will receive the same acupuncture treatment once a week for 6 weeks as a concurrent treatment. Changes in the 100-mm visual analogue scale (VAS) for low back pain after 6 weeks from baseline will be assessed as the primary outcome. Furthermore, the 100-mm VAS for radiating pain, Oswestry disability index (ODI), Roland–Morris disability questionnaire (RMDQ), EuroQol 5 Dimensions 5 Levels (EQ-5D-5L), global perceived effect (GPE), and deficiency syndrome of kidney index (DSKI) will be used to evaluate secondary outcomes. Outcomes will be assessed at baseline and at 3, 6, and 10 weeks after screening. For the safety evaluation, laboratory examinations including complete blood count, liver function test, renal function test, blood coagulation test, inflammation test, and urine analysis will be conducted before and after taking the medications.

**Discussion:**

The results of this trial will be used to establish clinical evidence regarding the use of Bosinji with acupuncture treatment in the treatment of patients with LHIVD.

**Trial registration number:**

NCT03386149 (clinicaltrials.gov) and KCT0002848 (Clinical Research Information Service of the Republic of Korea).

## 1 Introduction

A lumbar herniated intervertebral disc (LHIVD) is defined as a medical condition that causes compression and irritation of the dural sac and nerve roots due to displacement of the intervertebral disc in the lumbar region.^[1]^ As low back pain and radiating pain, which are the most common symptoms of LHIVD, greatly affect the daily life and working status of patients, pain relief is a major concern in the conservative treatment of patients with LHIVD.^[2,3]^

Herbal medicines have been used clinically for the treatment of LHIVD mainly in Asian countries, and there have been a number of reports of evidence of their effectiveness.^[4,5]^ Bosinji, the commercial name of *Ucha-Shinki-hwan* (*Goshajinkigan* in Japanese), which consists of 10 crude drugs, is one of the herbal formulations traditionally used for low back pain with radiating pain.^[6]^

Each component of Bosinji has shown several pharmacological effects in experimental studies; these effects may explain the mechanism of its effect on LHIVD. *Aconitum carmichaelii* exerts an anti-nociceptive effect by stimulating spinal kappa-opioid receptors via release of dynorphin,^[7]^ as well as a blood flow-increasing effect by promoting the production of nitric oxide.^[8]^ Catalpol, an iridoid glucoside found in *Rehmannia glutinosa*, also has an anti-nociceptive effect associated with modulation of neuroinflammation in the spinal cord in rats.^[9]^ Other components of Bosinji such as *Corni officinalis*,^[10]^*Alisma orientale*,^[11]^*Poria cocos*,^[12]^*Paeonia suffruticosa*,^[13]^ and *Cinnamomum cassia*,^[14]^ show anti-inflammatory effects.

Experimental research has shown Bosinji to be possessing properties similar to those of its individual components. Similar to *Aconitum carmichaelii*, Bosinji has an anti-nociceptive effect that involves the activation of kappa-opioid receptors, thereby reducing paresthesia,^[15]^ as well as vasodilating effect via increase in nitric oxide production.^[16]^ Nakanishi et al have reported that Bosinji suppresses the expression of tumor necrosis factor-α,^[17]^ which is a critical molecular mediator in the pathogenesis of radiating pain.^[1]^

Based on the mechanisms revealed in experimental studies, Bosinji have also been shown to be clinically effective in the treatment of LHIVD in observational studies.^[18,19]^ One randomized controlled trial (RCT) reported that the effectiveness of Bosinji was similar to that of nonsteroidal anti-inflammatory drugs (NSAIDs) for low back pain and paresthesia in the lower extremities; however, this trial carried many unclear risks of bias and methodological limitations, and hence, a definite conclusion could not be arrived at.^[20]^

For the clinical application of Bosinji in treating low back pain with radiating pain caused by LHIVD, reliable evidence from pragmatic RCT reflecting the actual clinical situation in Korean medical clinics where herbal medicine treatment is conducted with acupuncture treatment concurrently^[21]^ is required. Therefore, we will evaluate the equivalence of Bosinji to NSAIDs in concurrent with acupuncture using a rigorously designed, full-scale RCT protocol that includes assessments on pain, function, quality of life (QoL), and safety.

## 2 Methods

### 2.1 Trial design

This study is a multicenter, open-label, randomized, controlled, and equivalence trial with 2 parallel arms (1:1 ratio). The trial will be conducted at the Kyung Hee University Hospital at Gangdong (KHUHGD), Kyung Hee University Medical Center (KHUMC), Dongguk University Bundang Oriental Hospital (DUBOH), and Daegu Korean Medicine Hospital of Daegu Haany University (DKMHDHU). The efficacy and safety of Bosinji in patients with low back pain with radiculopathy due to LHIVD will be evaluated by comparison with loxoprofen, one of the NSAIDs.

This protocol has been approved by the Institutional Review Boards (IRBs) of the institutions (KHUHGD: KHNMCOH 2017-08-002, KHUMC: 170918-HR-039, DUBOH: 2017-0007, and DKMHDHU: DHUMC-D-17019). The trial has also been registered at clinicaltrials.gov, which is a website of the United States National Institutes of Health (registration number: NCT03386149), as well as in the Clinical Research Information Service of the Republic of Korea (registration number: KCT0002848). All research procedures comply with Korean Good Clinical Practice (KGCP) and the Declaration of Helsinki. The methodology was established in accordance with the Standard Protocol Items: Recommendation for Interventional Trials (SPIRIT)^[22]^ and with the revised Standards for Reporting Interventions in Clinical Trials of Acupuncture (STRICTA).^[23]^

### 2.2 Participants

The inclusion criteria for this study are as follows: age over 19 years; radiating pain consistent with abnormalities in the lumbar spine that are more severe than bulging, as shown on computerized tomography (CT) or magnetic resonance imaging (MRI)^[24,25]^; low back pain score between 40 and 80 points on the 100-mm pain visual analogue scale (VAS); and voluntary participation and provision of a signed informed consent form after a detailed explanation of the clinical trial has been provided.

Participants who have the following characteristics will be excluded:

1. congenital abnormalities or surgical history in the lumbar region;
2. red flag signs that may indicate cauda equina syndrome, such as bladder and bowel dysfunction or saddle anesthesia;
3. tumor, fracture, or infection in the lumbar region;
4. injection in the lumbar region within 1 week of the screening;
5. psychiatric disorder currently being treated, such as depression or schizophrenia;
6. liver function abnormality (aspartate transaminase [AST] or alanine transaminase [ALT] >100 U/L for men/70 U/L for women);
7. renal function abnormality (serum creatinine >2.0 mg/dL);
8. other diseases that could affect or interfere with therapeutic outcomes, including severe gastrointestinal disease, cardiovascular disease, hypertension, diabetes, renal disease, liver disease, or thyroid disorder;
9. contraindications for NSAIDs, including concurrent disease, hypersensitivity reaction, or other medication;
10. conditions for which acupuncture is contraindicated, for example, skin disease or hemostatic disorder (international normalized ratio >2.0 or taking anticoagulant);
11. pregnancy, breastfeeding or having pregnancy plans;
12. other condition for which herbal medicine treatment is contraindicated; and
13. participation in other clinical trials within 1 month of the screening.

### 2.3 Procedure

**Figure 1:**
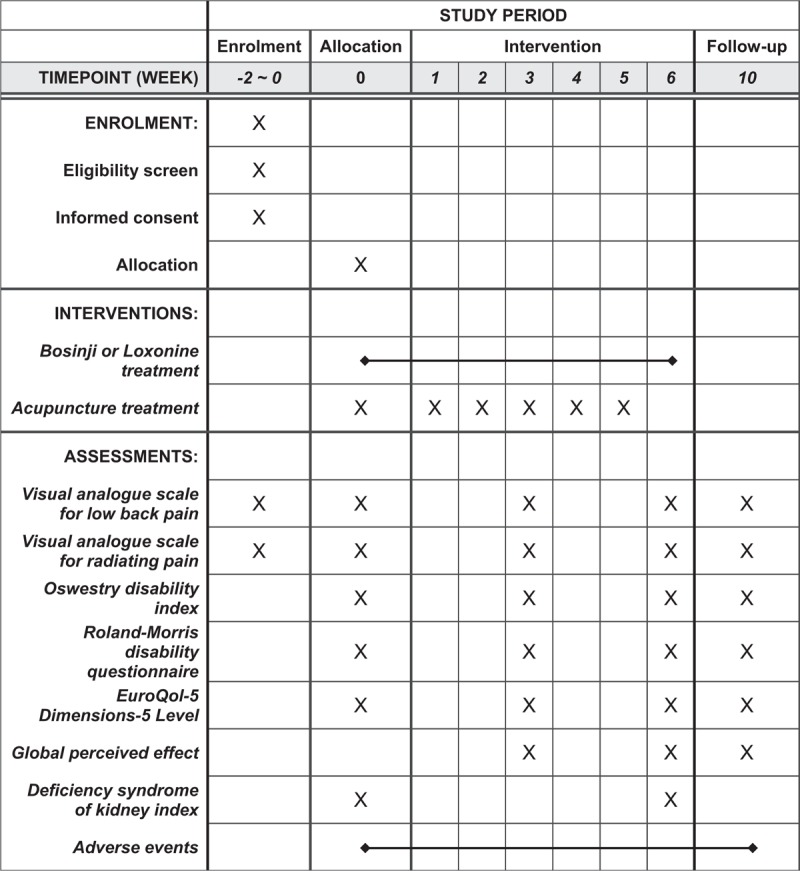
Standard Protocol Items: Recommendations for Interventional Trials (SPIRIT) figure.

A total of 74 participants with LHIVD will be recruited at four institutions, each of which will recruit an appointed number of patients (KHUHGD: 20, KHUMC: 18, DUBOH: 18, and DKMHDHU: 18). All participants who can read and write in Korean will be informed that they may voluntarily participate and that they can withdraw their consent at any stage. They will also be given essential information regarding the study protocol, including purpose, selection of participants, interventions by random allocation, schedule, expected benefits and risks, alternative treatment options, and confidentiality. Those who agree and sign the informed consent form will be screened through the assessment of demographics, medical history, present illness, vital sign, pregnancy test, and laboratory test. If they meet the eligibility criteria, the participants will be randomly allocated to the experimental group or control group. After random allocation, the assigned group will undergo a 6-week treatment, and assessment of outcome measure will be performed at 3, 6, and 10 weeks later according to schedule (Fig. 1).

### 2.4 Interventions

In the experimental group, 2.5 g of Bosinji granule (1.523 g of Bonsinji extract, Tsumura Co., Tokyo, Japan) will be orally administered 3 times a day at 30 minutes after every meal for 6 weeks. Bonsinji extract comprises a mixture of extract from the following 10 crude drugs in fixed proportions (Table 1). Bosinji is manufactured by Tsumura Co. in compliance with Good Manufacturing Practice standards. In the preparation phase, each crude drug undergoes macroscopic and microscopic examination. The extract and its components are also subjected to physicochemical tests. Bosinji granule is produced by a process involving decoction, separation, concentration, drying, and granulation. To ensure consistency and safety, multiple quality tests, including quantitative analysis of the components, microbial limits test, and residual pesticides test, are performed.

**Table 1:**
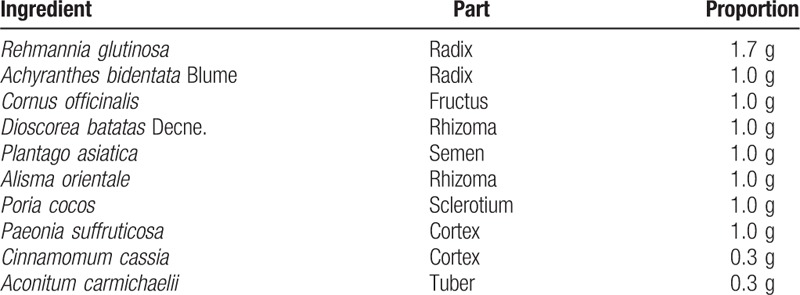
Composition of Bosinji extract.

In the control group, Loxonin tablet (60 mg of loxoprofen, Dong Wha Pharm Co., Ltd, Seoul, Korea) will be orally administered 3 times a day at 30 minutes after each meal for 6 weeks. Three weeks of medication will be provided at week 0 and week 3, and the remaining medication will be withdrawn to confirm the medication compliance.

### 2.5 Concurrent treatment

As a concurrent treatment, an identical acupuncture treatment will be conducted once a week for all participants regardless of group during the medication period. Acupuncture treatment will be performed on 20 predefined acupoints using 40 mm x 0.25 mm acupuncture needles according to STRICTA (Table 2). The selection of acupoints and details of the procedure have been modified from similar studies^[26,27]^ by a committee of Korean medical doctors (KMDs) experienced in LHIVD and acupuncture. Acupuncture treatment will be performed by KMDs who have completed or have been taking a specialist acupuncture and moxibustion course for at least 3 years. To maintain homogeneity of treatment among the participating centers, all practitioners will undertake a training course together in advance.

**Table 2:**
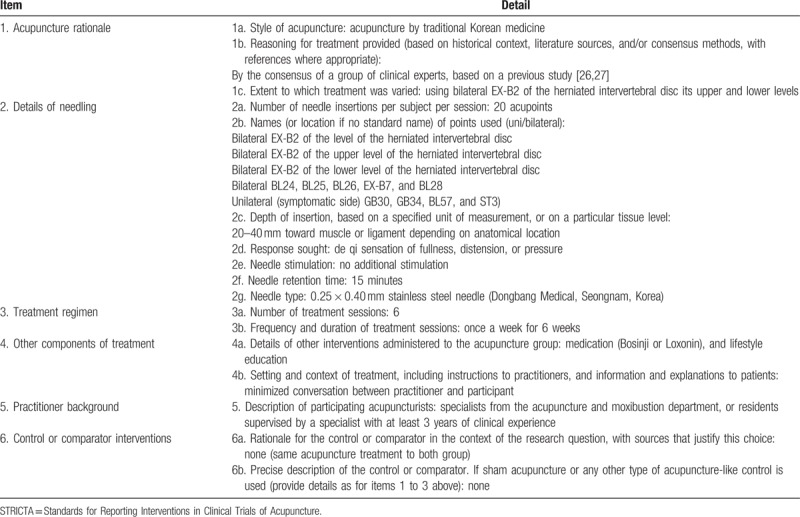
Details of acupuncture treatment using the STRICTA 2010 checklist.

### 2.6 Outcomes

All assessments will be performed according to schedule by blinded independent researchers who are not involved in the intervention. The primary outcome will be a change in VAS for low back pain. The secondary outcomes will be evaluated using the VAS for radiating pain, Oswestry disability index (ODI), Roland-Morris disability questionnaire (RMDQ), EuroQol 5 Dimensions 5 Levels (EQ-5D-5L), global perceived effect (GPE), and deficiency syndrome of kidney index (DSKI). Additionally, all adverse events will be recorded at each visit.

#### 2.6.1 Primary outcome measure

##### 2.6.1.1 100-mm VAS for low back pain

The intensity of low back pain will be evaluated using the 100-mm VAS at pre-trial screening and at weeks 0 (baseline), 3, 6 (primary endpoint), and 10 (follow-up sessions).^[28]^ Participants will be asked to record their pain intensity within the past week on a 100-mm linear scale (0, absence of pain; 100, worst pain imaginable). Changes from baseline after 6 weeks of treatment will be compared between the groups as the primary outcome.

#### 2.6.2 Secondary outcome measures

##### 2.6.2.1 100-mm VAS for radiating pain

The intensity of radiating pain in the lower extremities will also be assessed using a 100-mm VAS at pre-trial screening and at weeks 0, 3, 6, and 10. Participants will be asked to record their pain intensity within the past week on a 100-mm linear scale (0, absence of pain; 100, worst pain imaginable).

##### 2.6.2.2 Oswestry disability index (ODI)

Inability to function in daily life due to low back pain will be assessed using the ODI at weeks 0, 3, 6, and 10. The ODI questionnaire consists of 10 sections: pain, personal care, lifting, walking, sitting, standing, sleeping, sex life, social life, and traveling. Each question is scored from 0 to 5, and the total score is calculated as a percentage disability.^[29]^

##### 2.6.2.3 Roland-Morris disability questionnaire (RMDQ)

Physical disability due to low back pain will be assessed using the RMDQ at weeks 0, 3, 6, and 10. The RMDQ contains 24 sentences describing the discomfort that may occur with low back pain. Participants will need to check the sentences that would best describe their life on that day, and the score is based on the total number of sentences checked (from 0 to 24).^[30]^

##### 2.6.2.4 EuroQol 5 Dimensions 5 Levels (EQ-5D-5L)

The QoL and general health status will be assessed using the EQ-5D-5L at weeks 0, 3, 6, and 10. The EQ-5D-5L consists of the EQ-5D descriptive system and EQ-VAS. The EQ-5D descriptive system assesses the following five dimensions: mobility, self-care, usual activities, pain/discomfort, and anxiety/depression. Each dimension is rated from 1 to 5 (1, no problem; 2, slight problems; 3, moderate problems; 4, severe problems; and 5, extreme problems). The EQ-VAS scale can be used to assess a patient's current health status. It is a 20-cm scale numbered from 0 to 100 (0, the worst health they can imagine; 100, the best health they can imagine).^[31]^

##### 2.6.2.5 Global perceived effect (GPE)

The subjective change of symptom will be assessed using the GPE at weeks 3, 6, and 10. The participants will score their perceived change after treatment using a 7-point scale (1, worst ever; 2, much worse; 3, worse; 4, not improved and not worse; 5, slightly improved; 6, much improved; and 7, best ever).^[32]^

##### 2.6.2.6 Deficiency syndrome of kidney index (DSKI)

The deficiency syndrome of kidney (DSK), a concept of the Korean medical diagnosis system, will be assessed using DSKI at weeks 0 and 6. The DSKI shows the severity of the DSK by scoring the 12 DSK-related symptoms from 0 to 2 points (1, no symptom; 2, moderate; and 3, severe).^[33]^

### 2.7 Safety

To confirm the safety of the medications, the following laboratory tests will be carried out at the pre-screening point and at week 6 when the medication treatment is completed; complete blood count (white blood cells, red blood cells, hemoglobin, hematocrit, and platelet), liver function test (total protein, albumin, AST, ALT, alkaline phosphatase, total bilirubin, and γ-glutamyl transferase), renal function test (uric acid, blood urea nitrogen [BUN], and creatinine), fasting plasma glucose, prothrombin time, activated partial thromboplastin time, erythrocyte sedimentation rate, C-reactive protein, and urine analysis (color, specific gravity, pH, protein, glucose, ketone, urobilinogen, bilirubin, nitrite, blood, and white blood cell). Additionally, AST, ALT, BUN, and creatinine will be examined at week 3. For women of childbearing potential, a urine human chorionic gonadotropin test will be performed using a stick-type pregnancy tester, and they will be educated about the need for medically acceptable contraception during the study period.

At each visit, the researchers will measure the vital signs, such as blood pressure, pulse rate, and body temperature and collect information about the occurrence of adverse event (AE) and changes in the drug being taken. Information about expected AEs, as well as a contact number, will be given to the participants along with the informed consent form before the pre-trial screening. If AEs do occur, the principal investigator will evaluate the severity of the incident, as well as its relation to the interventions, and provide proper examination and treatment in accordance with the compensation rules. The progress of all AEs will be recorded in the case report form (CRF) and handled in accordance with the standard operation protocol (SOP) of the IRB and the regulation of KGCP.

### 2.8 Sample size

The sample size was calculated based on a previous similar study (assuming σ = 9.18, 
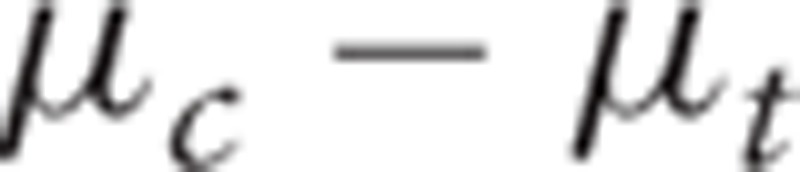
 = 10),^[20]^ and the equivalence margin for the primary outcome was set at 18.^[34]^ With a 0.05 significance level, 80% power, and 1:1 ratio, we calculated the adequate sample size using the following formula:
 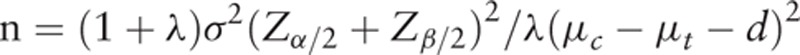


Finally, we determined that the sample size required in each group should be 37 participants by considering the dropout rate (20%) and medication compliance rate (95%) on the calculated value. The 74 participants will be recruited separately at the four research sites; each site will recruit the following numbers of participants: KHUHGD: 20, KHUMC: 18, DUBOH: 18, and DKMHDHU: 18.

### 2.9 Randomization and allocation concealment

A total of 74 participants will be randomly allocated to the experimental group or control group according to a block randomization procedure with a 1:1 ratio after stratifying them by institutions. The randomization sequence will be generated by an independent statistician using the package “blockrand” 1.3 of R (The R Foundation for Statistical Computing, Vienna, Austria). For allocation concealment, sealed, opaque envelopes containing random code will be sent to each institution. The clinical research coordinator will open the envelope and allocate participants to their groups after screening.

### 2.10 Blinding

The medications used in this study are in the form of tablets and granules and can easily be distinguished visually. Therefore, because it is impossible to achieve patient blinding, this research was designed as an open-label study. However, for objective analysis, evaluators and statisticians do not provide information on group assignments.

### 2.11 Statistical methods

The data will be corrected using the “last observation carried forward” method and then analyzed using the “intention-to-treat” principle. The independent *t* test and Chi-square test will be used to compare the differences in general characteristics between groups. As a primary outcome, changes in the 100-mm VAS for low back pain from baseline to the end of the end of the medication treatment will be compared between groups using an independent *t* test. Equivalence will be evaluated based on the equivalence margin of 18 mm and 95% confidence interval (CI). To compare the outcomes of each session with the baseline values, an analysis of covariance will be used. Trends over time and time-by-treatment interactions will be analyzed using a repeated measures analysis of variance. All statistical analyses will be performed using PASW statistics 18 for Windows, and the statistical significance level will be set at .05.

### 2.12 Data collection and management

All research data will be collected and double-checked by two independent researchers based on source documents including the CRF. All sensitive information obtained from the trial will be confidentially preserved, and personally identifiable information will be discarded after a certain period, in accordance with the SOP. All researchers will be given training in protecting the privacy of participants.

### 2.13 Quality control

To maintain the quality of the trial, the study procedure and documents will be periodically monitored by the Korean Medicine Clinical Trial Center.

## 3 Discussion

Bosinji is a granule-shaped herbal medicine derived from *Ucha-Shinki-hwan,* which is described in a medical book called “*Je-Saeng-Bang*” written in the 13th century.^[6]^ Due to the characteristic of this herbal formulation composed of 10 kinds of herbs, *Ucha-Shinki-hwan* has been reported to have beneficial effects on various diseases including chemotherapy-induced neuropathy,^[35–37]^ diabetic neuropathy,^[38]^ and overactive bladder.^[39,40]^ In addition, its effect on low back pain with radiating pain was reported in an RCT; however, there were many unclear risks of bias in random sequence generation, allocation concealment, outcome assessment blinding, and incomplete outcome data. In addition, since the outcome measurement was only limited to pain, it is not enough to confirm the definite evidence due to the low quality of the report.^[20]^

To confirm the efficacy of Bosinji compared with NSAIDs for low back pain and radiculopathy caused by LHIVD, this RCT was designed in a methodologically rigorous, full-scale setting. The indicators for pain, function, QoL, and safety will be comprehensively evaluated, and DSKI will be assessed to reflect the diagnostic conception of traditional Korean medicine. Because *Ucha-Shinki-hwan* was originally designed to treat a group of complex symptoms called DSK,^[6]^ not a single specific symptom, it is necessary to verify the relation between the therapeutic effect and traditional diagnostic system.

Loxoprofen, used as an active control intervention in this study, is one of the NSAIDs in the propionic acid group.^[41]^ The clinical benefit of NSAIDs for LHIVD remains controversial; however, they are most commonly used for conservative treatment of LHIVD because inflammatory signaling plays an important role in the production of nerve pain.^[1,2]^ Therefore, NSAIDs have been used as a control intervention in studies attempting to identify a new therapeutic agent for LHIVD.^[42]^ Although patient blinding is not possible due to the differences in the shape of medications, bias can be minimized through blinding of assessors and statisticians.

As a concurrent treatment, acupuncture will be practiced to reflect the actual clinical situation in Korea. While about 90% of patients who visit Korean medical institutions due to low back pain choose acupuncture as a primary treatment, about 30% of patients opt for herbal medicine treatment.^[21]^ Therefore, evaluating the effects of herbal medicine along with acupuncture may help determine the practical usefulness.

The results of this study will suggest clinical evidence comparing Bosinji with NSAIDs by providing data about the changes in various measurements from the rigorously conducted trial. These findings will help clinicians to utilize Bosinji with acupuncture as a therapeutic option for patients with LHIVD.

## Author contributions

**Conceptualization:** Byung-Kwan Seo.

**Funding acquisition:** Byung-Kwan Seo.

**Methodology:** Eun-Jung Kim, Dongwoo Nam, Hyun-Jong Lee, Jae-Soo Kim, Yeon-Cheol Park, Yong-Hyeon Baek, Sang-Soo Nam, Byung-Kwan Seo.

**Project administration:** Bonhyuk Goo, Sung-Jin Kim, Eun-Jung Kim, Dongwoo Nam, Hyun-Jong Lee, Jae-Soo Kim, Byung-Kwan Seo.

**Supervision:** Byung-Kwan Seo.

**Writing – original draft:** Bonhyuk Goo, Sung-Jin Kim.

**Writing – review & editing:** Eun-Jung Kim, Dongwoo Nam, Hyun-Jong Lee, Jae-Soo Kim, Yeon-Cheol Park, Yong-Hyeon Baek, Sang-Soo Nam, Byung-Kwan Seo.
